# High‐Order Multipolar Excitations Governing Circular Dichroism in an All‐Dielectric Chiral Metasurface

**DOI:** 10.1002/nap2.70153

**Published:** 2026-05-28

**Authors:** Chien‐Feng Chien, Hsiu‐Ping Su, Yu‐Tsung Lin, Shu‐Fei Ho, Pin Chieh Wu

**Affiliations:** ^1^ Department of Photonics National Cheng Kung University Tainan Taiwan; ^2^ Center for Quantum Frontiers of Research & Technology (QFort) National Cheng Kung University Tainan Taiwan; ^3^ Meta‐nanoPhotonics Center National Cheng Kung University Tainan Taiwan

**Keywords:** circular dichroism, dielectric metasurfaces, multipolar resonances, optical chirality

## Abstract

Chiral metasurfaces provide a versatile platform for tailoring polarization‐dependent light–matter interactions with strong field confinement. Here, we report an all‐dielectric asymmetric gammadion metasurface that exhibits pronounced circular dichroism (CD) in the visible spectral range. By breaking the in‐plane mirror symmetry of the unit cell, strong polarization‐selective resonances are induced under circularly polarized illumination. Numerical simulations and experiments reveal two distinct CD resonances near 652 and 735 nm with maximum absolute CD values exceeding 50% and 40%, respectively. Electromagnetic multipole decomposition shows that the strong CD at 652 nm originates from selective excitation of high‐order electric and magnetic modes under right‐handed circularly polarized illumination leading to a large imbalance in multipolar scattering and strongly localized chiral near fields. In contrast, the weaker CD at 735 nm arises from a more symmetric and reduced multipolar excitation landscape. Angle‐resolved analyses further demonstrate that the quadrupole‐dominated resonance maintains robust CD under oblique incidence, whereas the simpler multipolar excitation at 735 nm yields a more angle‐insensitive but moderate CD response. These results establish a physical link between structural asymmetry, multipolar excitation, near‐field optical chirality, and angle‐resolved chiroptical behavior, providing practical design guidelines for low‐loss dielectric metasurfaces with robust chiroptical responses.

## Graphical abstract

An all‐dielectric asymmetric gammadion metasurface exhibits visible circular dichroism governed by helicity‐selective multipolar excitations. Quadrupole‐dominated and weaker mixed‐mode resonances are identified, and their distinct near‐field optical chirality and angular responses are revealed, establishing a direct link between structural asymmetry, multipolar redistribution, and chiroptical behavior.

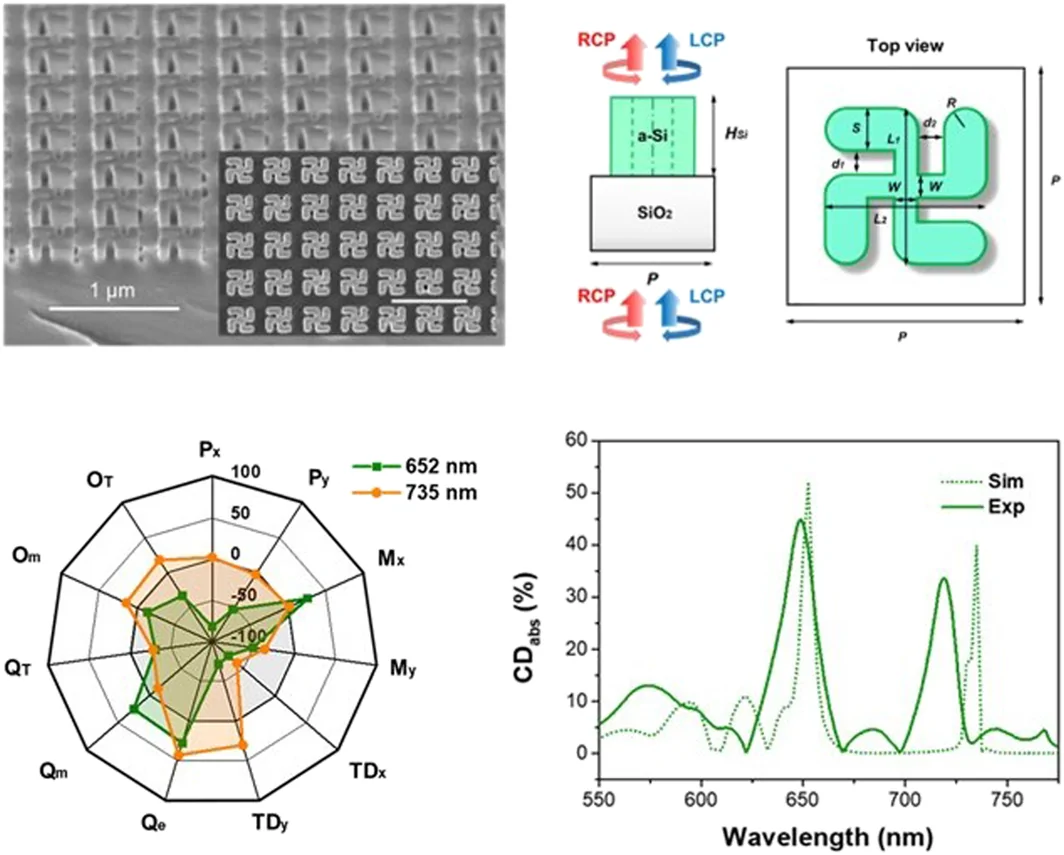

## 1 Introduction

Circular dichroism (CD), defined as the differential optical response of a system to left‐ and right‐handed circularly polarized light, constitutes a fundamental manifestation of chiral light–matter interactions [1, 2]. It has long served as a critical tool in molecular spectroscopy, enantiomeric discrimination, and polarization‐sensitive photonic technologies [3, 4]. However, in natural chiral materials, CD signals are typically weak due to limited intrinsic chirality and the inefficient coupling between light and matter at subwavelength scales [5]. Over the past decade, metasurfaces (planar arrays of artificially engineered nanostructures) [6, 7, 8] have emerged as a powerful platform for overcoming these limitations by enabling precise control over electromagnetic fields through structural symmetry breaking and resonant enhancement [9, 10, 11].

Among various metasurface platforms, all‐dielectric chiral metasurfaces [12, 13, 14] have attracted increasing attention owing to their low optical loss, high refractive index contrast, and ability to sustain sharp resonances with strong field confinement. Unlike plasmonic systems, dielectric metasurfaces support a rich hierarchy of electromagnetic multipolar modes, including not only electric and magnetic dipoles but also higher‐order excitations such as electric and magnetic quadrupoles and toroidal modes [15, 16, 17]. These high‐order multipolar resonances play a crucial role in shaping both near‐field and far‐field optical responses, offering new degrees of freedom for tailoring chiroptical effects. In particular, symmetry‐broken dielectric structures can enable polarization‐selective excitation of specific multipolar channels, giving rise to enhanced CD and localized chiral near‐fields.

Recent studies have demonstrated that the excitation and interference of multipolar modes can be engineered to amplify chiroptical responses [18, 19, 20], generate high‐quality‐factor resonances [12, 21, 22] and even approach quasi‐bound states in the continuum [23, 24]. Rigorous theoretical tools [25], including electromagnetic multipole decomposition, Mie theory, and quasinormal mode analysis, have provided valuable insights into the modal origins of chiral optical phenomena. Nevertheless, a comprehensive physical picture linking high‐order multipolar excitations to optical chirality enhancement and robust CD responses remains incomplete, particularly for all‐dielectric metasurfaces operating in the visible regime and under oblique illumination. In many reported systems, CD enhancement is often attributed qualitatively to structural chirality, whereas the specific modal contributions and their polarization‐dependent excitation pathways are not fully resolved.

In this work, we investigate the chiroptical response of an all‐dielectric asymmetric gammadion metasurface composed of amorphous silicon (a‐Si), with a particular focus on the role of high‐order multipolar resonances in governing CD and optical chirality. By performing a systematic electromagnetic multipole decomposition [15, 26], we show that the pronounced CD observed at visible wavelengths originates from polarization‐selective excitation of electric and magnetic high order modes enabled by broken in‐plane mirror symmetry. Through detailed analysis of near‐field electric and magnetic distributions, we further demonstrate that these high‐order modes generate strongly localized chiral near‐fields leading to a substantial enhancement of optical chirality. Moreover, we examine the angular dependence of the chiroptical response and reveal that the metasurface maintains a robust CD over a wide range of incident angles, reflecting the combined influence of intrinsic and extrinsic chirality supported by the multipolar resonances. Although planar dielectric gammadion metasurfaces with strong chiro‐optical responses have previously been reported, the present work addresses a different physical regime and question. For example, in the TiO_2_ gammadion/waveguide platform [14], the main resonance was associated with dominant higher‐order radiation, especially toroidal‐quadrupole and magnetic‐octupole contributions, in an optically thick diffraction‐assisted configuration. By contrast, our asymmetric a‐Si gammadion metasurface is used here to elucidate how broken in‐plane mirror symmetry redistributes the helicity‐dependent excitation among dipolar, quadrupolar, and toroidal channels across two visible CD resonances. Therefore, this work lies not simply in invoking higher‐order multipoles, but in establishing an explicit link between structural asymmetry, multipolar redistribution, and localized optical chirality enhancement of the chiroptical response. Our results elucidate the fundamental multipolar mechanisms underlying chiroptical phenomena in dielectric metasurfaces and provide clear physical guidelines for designing polarization‐selective nanophotonic devices with enhanced and resilient chiral responses.

## 2 Results and Discussion

Figure 1 summarizes the structural design, fabrication quality, and basic chiroptical response of the proposed all‐dielectric asymmetric gammadion metasurface. As shown in Figure 1A, the metasurface consists of a‐Si nanostructures arranged in a periodic square lattice on a SiO_2_ substrate. The gammadion geometry is intentionally designed with unequal arm lengths and asymmetric air gaps, thereby breaking the in‐plane mirror symmetry while preserving a compact subwavelength footprint. This structural asymmetry is essential for enabling polarization‐dependent light–matter interactions and generating intrinsic optical chirality under normal incidence. The use of high‐refractive‐index a‐Si allows the metasurface to support strong electromagnetic resonances in the visible spectral range, providing a suitable platform for multipolar mode engineering.

**FIGURE 1:**
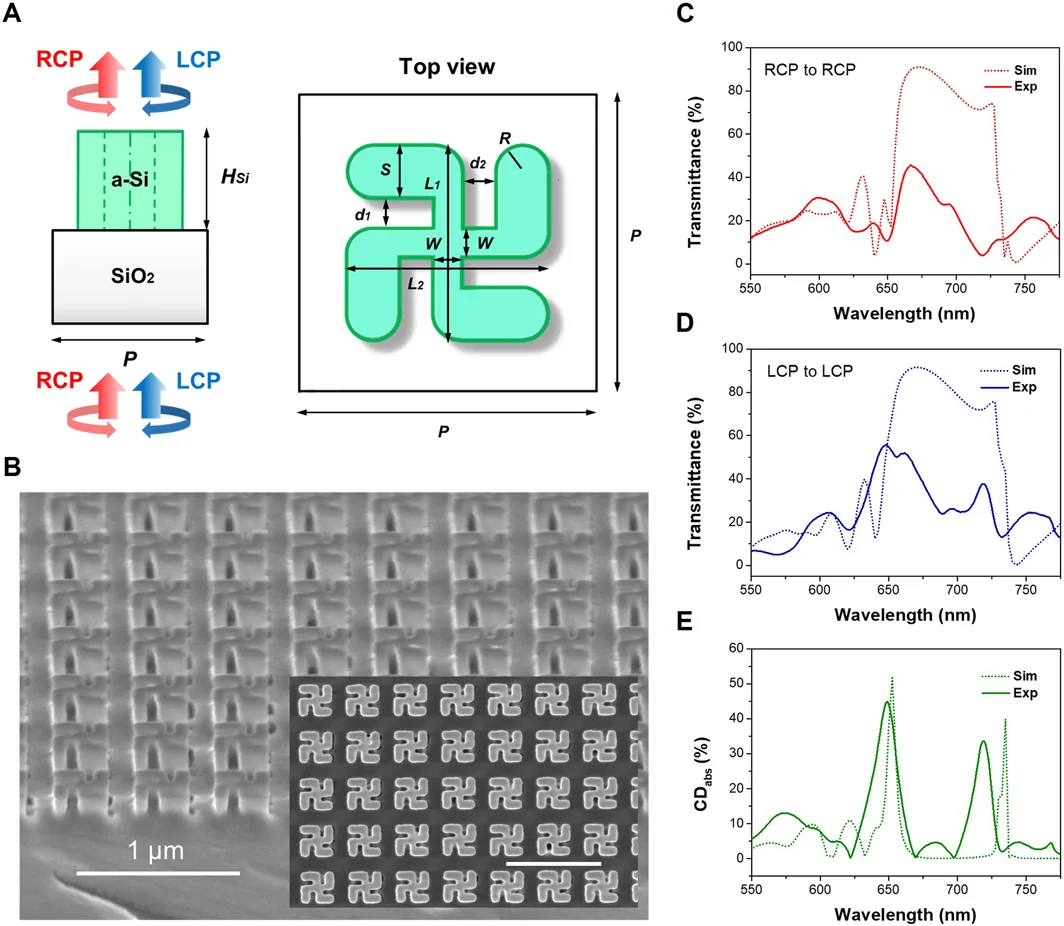
(A) Schematic illustration of the asymmetric gammadion metasurface composed of a‐Si on a SiO_2_ substrate, together with the definition of geometric parameters. The metasurface has a periodicity *P* = *P*_*x*_ = *P*_*y*_ = 500 nm, a‐Si layer thickness *H*_Si_ = 310 nm, arm lengths *L*_1_ = 330 nm and *L*_2_ = 340 nm, air gap *d*_1_ = 50 nm and *d*_2_ = 55 nm, and linewidth *W* = 50 nm, arm width *S* = 90 nm, and *R* = *S*/2. (B) SEM images of the fabricated metasurface array. The central panel shows a tilted‐view image illustrating the uniformity and vertical profile of the nanostructures, whereas the right panel presents a top‐view image of the periodic gammadion unit cells. The scale bar corresponds to 1 μm. (C, D) Simulated transmission spectra of the metasurface under RCP and LCP illumination, respectively. (E) Absolute circular dichroism (CD_abs_) spectrum calculated from the results shown in panels (C) and (D).

The fabrication quality of the metasurface is verified by the scanning electron microscopy (SEM) images shown in Figure 1B. The tilted‐view SEM image confirms the uniformity of the nanostructure array and the well‐defined vertical sidewalls of the a‐Si elements, whereas the top‐view image reveals high fidelity of the asymmetric gammadion geometry across the entire patterned area. Subsequently, we investigated the transmittance and CD properties of the all‐dielectric asymmetric gammadion metasurface under normal incidence of right‐handed circularly polarized (RCP) and left‐handed circularly polarized (LCP) light.

Figure 1C,D present the simulated transmission spectra under RCP and LCP illumination, respectively. The discrepancies between simulation and experiment are mainly attributed to fabrication induced dimensional deviations, such as linewidth and corner rounding. In addition, because the optical measurements were performed using a 10 × objective lens with a numerical aperture of 0.26, the incident beam contains finite oblique angle components rather than an ideal normally incident plane wave. Additional simulations under 15° oblique incidence show better agreement with the experimental spectra (see Supporting Information S1: Figure S5), indicating that both geometric deviations and finite angle illumination contribute to the observed differences. It is notable that the transmittance under LCP illumination is significantly higher than that under RCP, indicating stronger resonant excitation for RCP incidence, which will be further analyzed in subsequent sections. Pronounced differences between the two polarization states are observed at specific wavelengths, indicating a strong polarization‐selective resonant response induced by the asymmetric geometry. The corresponding absolute CD (CD_abs_) spectrum, shown in Figure 1E, exhibits distinct CD features in the visible regime, confirming the presence of significant chiroptical activity in the metasurface. Here, the CD formula is determined by the absorption difference (*A*_LCP_ and *A*_RCP_) or transmittance difference (*T*_LCP_ and *T*_RCP_) between incident LCP and RCP as follows [27]:

(1) $$\text{CD}=A_{\text{LCP}}-A_{\text{RCP}}=T_{\text{RCP}}-T_{\text{LCP}},$$

(2) $${\text{CD}}_{\text{abs}}=\left|\text{CD}\right|.$$

The simulations predict pronounced CD_abs_ peaks at 652 and 735 nm, reaching values of 52.1% and 40%, respectively. These results are in good agreement with experimental observations, where CD_abs_ peaks of 44.9% at 649 nm and 33.65% at 719 nm are recorded. Furthermore, comparative analysis with symmetric structures reveals that, under C_2_ symmetry, a single CD_abs_ peak emerges near 652 nm (as shown in Supporting Information S1: Figure S2). Upon breaking the symmetry to C_4_ symmetry [28], an additional peak appears at 735 nm, demonstrating the tunability of optical chirality through structural asymmetry. These results demonstrate that the asymmetric dielectric gammadion structure effectively converts structural chirality into measurable far‐field optical chirality. Additional polarization‐resolved measurements confirm that the cross‐polarized transmission components are much weaker than the copolarized ones in the present design (see Supporting Information S1: Figure S3). Therefore, including them in the CD_abs_ calculation [29] leads mainly to a quantitative change in amplitude, whereas the main resonance positions and physical interpretation remain unchanged. In the following sections, we elucidate the physical origin of these CD_abs_ resonances by analyzing the underlying multipolar excitations and their associated near‐field chiral characteristics.

To elucidate the physical origin of the pronounced CD_abs_ observed in the asymmetric gammadion metasurface, we perform an electromagnetic multipole decomposition of the scattered fields under circularly polarized illumination. This method is adopted here because it directly links the full‐wave optical response to the underlying electric, magnetic, and toroidal multipolar scattering channels, allowing us to identify the helicity‐dependent modal contributions responsible for the two observed CD_abs_ resonances. Figure 2A shows the wavelength‐dependent multipolar contributions under RCP excitation. At the CD_abs_ resonance wavelength of 652 nm, the scattering response is dominated by high‐order multipolar modes, most notably the electric quadrupole (*Q*_e_) and magnetic quadrupole (*Q*_m_), whereas the contributions from conventional electric and magnetic dipole modes (*P*_*x*_, *P*_*y*_, *M*_*x*_, and *M*_*y*_) remain comparatively weak. This clear dominance of quadrupolar modes indicates that the resonance at this wavelength is governed primarily by higher order multipolar scattering rather than dipolar resonances. Such high order modes are known to support stronger electromagnetic energy confinement within the nanostructure, which is consistent with the reduced transmittance and enhanced light–matter interaction observed at this resonance. In particular, the strong *Q*_e_ contribution suggests a highly localized electric field distribution inside the asymmetric gammadion geometry, a feature that will be directly visualized through near‐field analysis in Figure 3. To examine the possible interaction between adjacent meta‐units, we further analyzed the electric‐field distributions in a metasurface array at the two representative resonance wavelengths (refer to Supporting Information S1: Figure S8). In both cases, the electric fields remain predominantly confined within each individual gammadion element, whereas the field intensity in the regions between neighboring units is weak and shows no significant overlap. These results indicate that the resonant response is mainly localized to each meta‐unit and is governed by effective Mie‐type resonances of the high‐index dielectric structure. Therefore, although the lattice period is not deeply subwavelength over the entire spectral range, the observed chiroptical response is still dominated primarily by the polarization‐selective resonances of the individual meta‐units, whereas inter‐element coupling appears to be minor.

**FIGURE 2:**
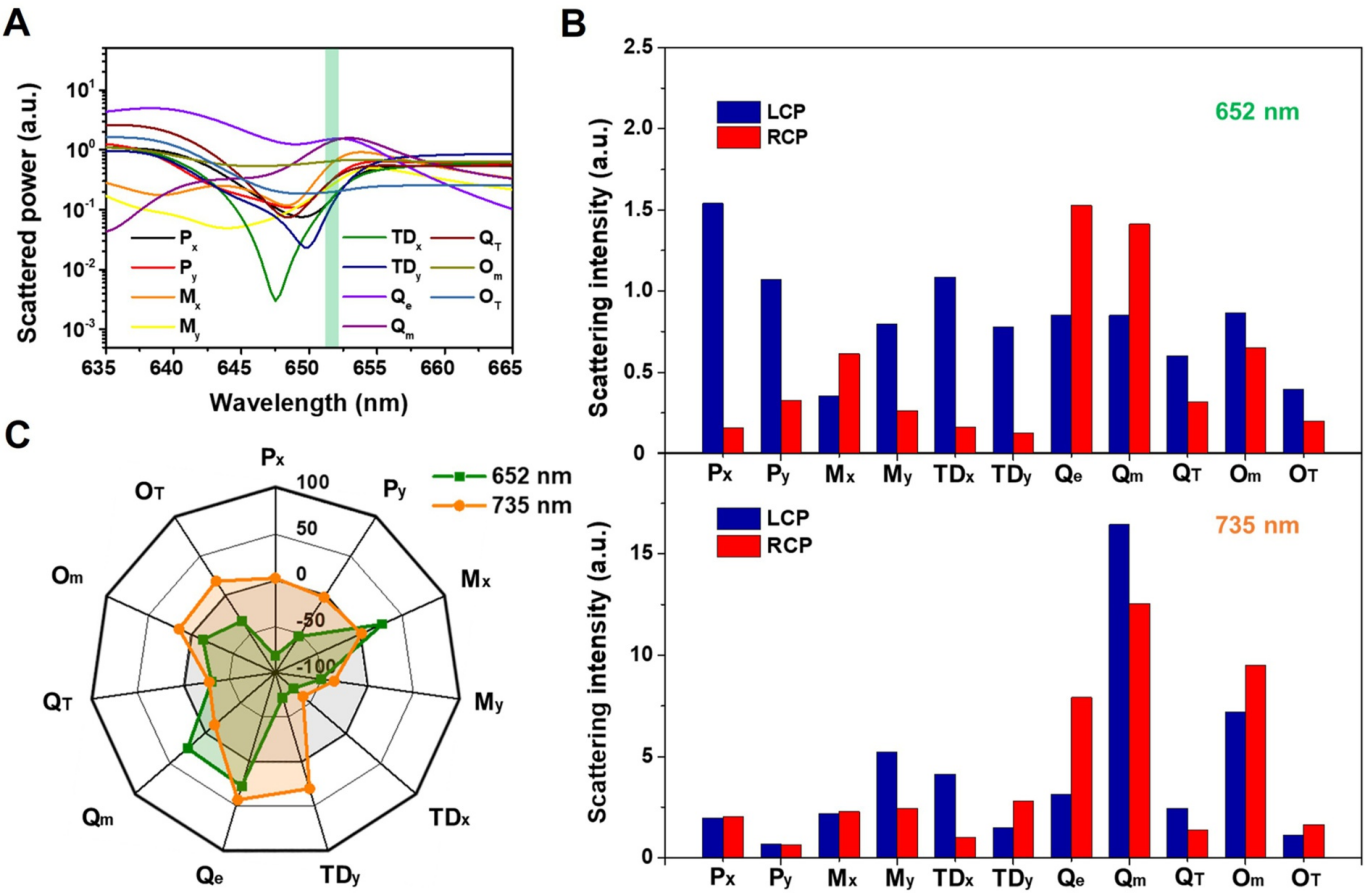
(A) Electromagnetic multipole decomposition of the scattered power from the all‐dielectric a‐Si asymmetric gammadion metasurface under RCP illumination. The shaded region marks the wavelength corresponding to the pronounced circular dichroism peak at 652 nm. Multipole components are defined as follows: *P*_*x*_ and *P*_*y*_, *x*‐ and *y*‐components of electric dipole; *M*_*x*_ and *M*_*y*_, *x*‐ and *y*‐components of magnetic dipole; TD_*x*_ and TD_*y*_, *x*‐ and *y*‐components of toroidal dipole; *Q*_e_, electric quadrupole; *Q*_m_, magnetic quadrupole; *Q*_T_, toroidal quadrupole; *O*_m_, magnetic octupole; and *O*_T_, toroidal octupole. (B) Bar charts comparing the scattering intensities of individual multipolar components under normal incidence for RCP and LCP illumination at wavelengths of 652 nm (top panel) and 735 nm (bottom panel) (C) Radar chart illustrating the relative differences in multipolar scattering contributions between RCP and LCP illumination at 652 and 735 nm.

**FIGURE 3:**
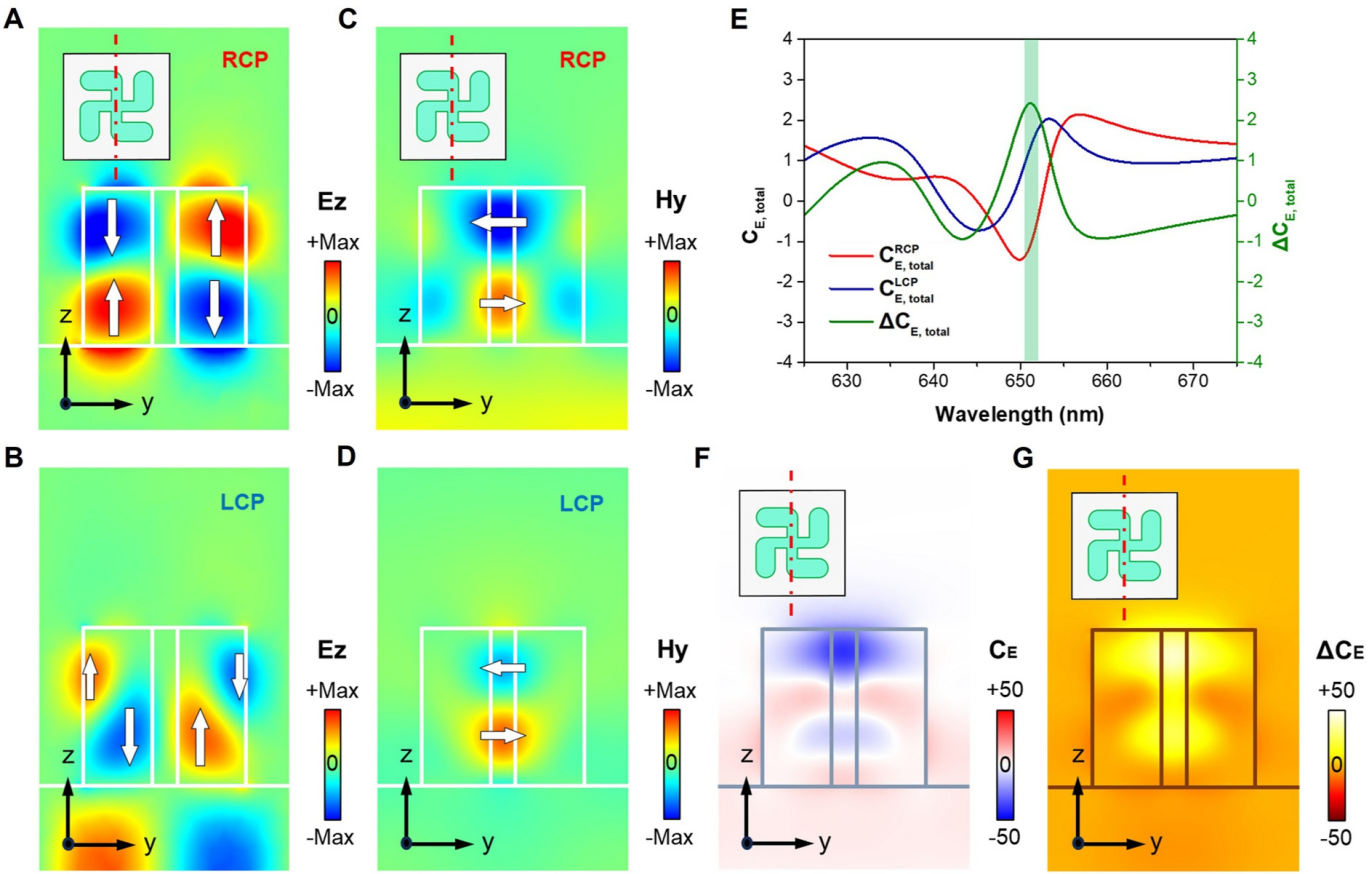
(A, B) Simulated *z*‐component of the induced electric field (*E*_*z*_) under RCP and LCP illumination, respectively, at a wavelength of 652 nm. (C, D) Corresponding *z*‐component of the induced magnetic field (*H*_*z*_) under RCP and LCP illumination at the same wavelength. The red dashed line in the inset schematics indicates the cross‐sectional plane used for visualizing the field distributions, whereas the white arrows denote the directions and relative phases of the induced electric or magnetic fields. (E) Spectral dependence of the total optical chirality enhancement (*C*_E, total_) under RCP and LCP illumination, together with the differential total optical chirality enhancement (Δ*C*_E, total_), within the wavelength range from 625 to 675 nm. The shaded region highlights the resonance wavelength at which the maximum chirality contrast occurs. (F) Spatial distribution of the optical chirality enhancement (*C*_E_) at 652 nm under RCP illumination. (G) Spatial distribution of the differential optical chirality enhancement (Δ*C*_E_) at 652 nm.

The polarization‐dependent nature of the multipolar excitations is further clarified by comparing the scattering intensities under RCP and LCP illumination. As summarized in Figure 2B, at 652 nm, the metasurface exhibits a pronounced asymmetry in modal excitation between the two polarization states. Under RCP illumination, the scattering response is dominated by electric and magnetic quadrupole modes (*Q*_e_ and *Q*_m_) accompanied by moderate contributions from the magnetic dipole along the *x* direction (*M*_*x*_) and the magnetic octupole (*O*_m_). In contrast, LCP illumination preferentially excites different modal channels, including electric dipole and toroidal dipole components, reflecting a strong polarization‐selective coupling enabled by the broken in‐plane mirror symmetry of the metasurface. This substantial contrast in modal composition directly leads to a large differential scattering response and gives rise to the pronounced CD observed at this wavelength. In contrast, at the longer wavelength resonance around 735 nm, where the CD_abs_ amplitude is comparatively weaker, the multipolar excitation profiles under RCP and LCP illumination become much more similar. As shown in the lower panel of Figure 2B, both polarization states excite a comparable set of modes, including electric dipoles, the magnetic dipole along the *x* direction, and toroidal octupole components. The absence of a dominant polarization‐selective high‐order mode at this wavelength reduces the imbalance between RCP and LCP scattering resulting in a weaker CD_abs_ response.

These trends are quantitatively summarized in the radar chart shown in Figure 2C, which presents the polarization‐dependent differences in scattering intensity for each multipolar component at 652 and 735 nm. The relative difference in multipolar scattering *S*_*r*_ is defined as follows:

(3) $$S_{r}=100\%\times \frac{I_{\text{RCP}}-I_{\text{LCP}}}{I_{\text{RCP}}+I_{\text{LCP}}}.$$

*I*_m_ denotes the scattering intensity of a given multipolar component under incident polarization m, where *m* = RCP or LCP. *S*_*r*_ denotes the normalized relative difference of a given multipolar scattering contribution under opposite circularly polarized excitations. The symbol *r* denotes the corresponding multipolar radiation channel considered in the decomposition, such as the electric dipole, magnetic dipole, toroidal dipole, electric quadrupole, or magnetic quadrupole contribution. At the CD_abs_ resonance wavelength of 652 nm, the radar chart reveals a highly asymmetric distribution of multipolar scattering strengths between the two circular polarizations. In particular, the *M*_*x*_, *Q*_e_, and *Q*_m_ components exhibit large positive contrasts, whereas the *P*_*x*_, *P*_*y*_, TD_*x*_, and TD_*y*_ components show pronounced negative contrasts, indicating a strong redistribution of scattering strength between RCP and LCP excitation. This pronounced imbalance in the multipolar scattering landscape directly reflects the strong polarization selectivity of the metasurface and constitutes the primary physical origin of the large CD_abs_ observed at this wavelength. Notably, this asymmetry is not confined to a single resonance but extends across multiple multipolar channels, underscoring that the observed CD_abs_ arises from a collective redistribution of scattering contributions among chiral‐sensitive modes rather than from an isolated multipolar excitation.

By contrast, at 735 nm, the relative differences across most multipolar components remain comparatively small as evidenced by the more symmetric radar pattern. The scattering strengths of the dominant modes under RCP and LCP illumination are of similar magnitude indicating that the metasurface responds to both helicities in a nearly equivalent manner at this wavelength. Nevertheless, certain modes, such as *Q*_e_, TD_*y*_, and *Q*_T_, still exhibit non‐negligible differences in $S_{r}$, giving rise to an observable but weaker CD_abs_ signal in the spectrum. This comparison between the two resonances highlights that the magnitude of CD is directly governed by the degree of asymmetry in the multipolar scattering distribution between opposite circular polarizations. It should be emphasized that the presence of high order multipolar excitations does not by itself guarantee a maximal CD_abs_ response. In the present metasurface, the role of the high order modes is to define the modal origin and helicity selectivity of the resonance. The absolute CD magnitude is instead determined by the degree of imbalance among the radiative multipolar channels excited by opposite helicities. At 652 nm, RCP selectively excites strong electric and magnetic quadrupole contributions, whereas LCP still couples to non‐negligible dipolar and toroidal channels, so the unfavored helicity is not completely suppressed. As a result, the differential transmission remains moderate rather than approaching unity. At 735 nm, the multipolar compositions under RCP and LCP become more similar, leading to a smaller helicity‐dependent imbalance and hence a weaker CD_abs_ response. In addition, the achievable CD_abs_ in the present platform is also influenced by the intrinsic material loss of a‐Si in the visible range. Although a‐Si provides a high refractive index favorable for supporting strong visible resonances, its extinction coefficient remains non‐negligible at these wavelengths. This material absorption reduces the resonance quality and weakens the buildup and interference of helicity‐selective multipolar channels, thereby limiting the peak CD_abs_. To verify this effect, we additionally performed simulations with a reduced imaginary part of the refractive index and found that the CD_abs_ increases when the extinction coefficient is lowered (refer to Supporting Information S1: Figure S6). This result confirms that the moderate CD_abs_ values in the present structure are governed not only by the multipolar excitation landscape but also by the optical loss of the dielectric material platform.

Figure 3 provides a direct visualization of the near‐field electromagnetic responses and optical chirality distributions that originate from the polarization‐dependent multipolar excitations identified in Figure 2. Figure 3A–D show the simulated z‐components of the induced electric and magnetic fields at the CD_abs_ resonance wavelength of 652 nm under RCP and LCP illumination. Consistent with the multipole decomposition results in Figure 2, RCP illumination gives rise to pronounced high‐order multipolar field patterns. In particular, the electric field distribution in Figure 3A exhibits two vertically aligned lobes with opposite phases across the structure, a hallmark of a dominant electric quadrupole *Q*_e_ resonance oriented along the *z* direction. Correspondingly, the magnetic field in Figure 3C displays multiple nodal features along the *y* direction, which is characteristic of a magnetic quadrupole Q_m_–type mode. In contrast, under LCP illumination (Figure 3B,D), the induced electric and magnetic fields are relatively weaker and exhibit distinctly different spatial distributions. The absence of well‐defined quadrupolar field patterns reflects the suppression of *Q*_e_ and *Q*_m_ modes under LCP excitation, in agreement with the multipolar analysis in Figure 2, where LCP preferentially couples to electric dipole and toroidal dipole channels. This pronounced asymmetry in near‐field intensity and modal composition between RCP and LCP excitation directly mirrors the far‐field multipolar imbalance and confirms the polarization‐selective nature of the asymmetric metasurface.

To quantitatively assess the resulting chiral near‐field enhancement, we evaluate the total optical chirality energy *C*_total_ defined as the volume integral of the optical chirality density *C*_E_ over the entire metasurface:

(4) $$C_{\text{total}}=\int C_{E}\;dV.$$

By normalizing this quantity to the optical chirality energy of an incident circularly polarized plane wave within the same volume *C*_CPL_, we obtain the total optical chirality enhancement:

(5) $$C_{E,\text{total}}=\frac{C_{\text{total}}}{C_{\text{CPL}}}.$$

This dimensionless ratio quantifies how efficiently the metasurface amplifies chiral near‐fields relative to the input CPL. Furthermore, the differential total optical chirality enhancement, defined as $\Delta C_{E,\text{total}}=C_{E,\text{total}}^{\text{RCP}}-C_{E,\text{total}}^{\text{LCP}}$, provides a direct measure of the metasurface's ability to discriminate between opposite helicities in terms of localized chiral energy generation. The superscript denotes the incident polarization of incident light. As shown in Figure 3E, both RCP and LCP illumination produce pronounced optical chirality enhancement in the vicinity of the resonance at 652 nm. Notably, the two curves exhibit opposite signs due to the opposite handedness of the excited near fields, resulting in a large magnitude of $\Delta C_{E,\text{total}}$ that peaks at the same wavelength. This behavior directly correlates with the strong *Q*_e_‐ and *Q*_m_‐dominated excitation under RCP illumination and their suppression under LCP illumination as revealed in Figure 2. The spatial origins of this enhancement are further elucidated in Figure 3F,G, which show the distributions of *C*_E_ and Δ*C*_E_ at 652 nm. The chiral near fields are strongly localized in regions associated with the quadrupolar field lobes, particularly in the upper and lower parts of the unit cell. Under RCP illumination, these regions exhibit significantly stronger and more confined optical chirality compared to the LCP case, leading to a pronounced differential enhancement. This clear correspondence between multipolar field distributions and localized optical chirality confirms that the large CD observed in this system arises from the selective excitation of high‐order multipolar resonances that generate strongly chiral near fields.

To further elucidate the robustness of the chiroptical response under realistic illumination conditions, we analyze the transmittance and absolute circular dichroism CD_abs_ of the asymmetric gammadion metasurface under oblique incidence. Figure 4A,B show the angle‐resolved transmittance maps under RCP and LCP illumination in the spectral region around the primary resonance near 652 nm, with the incident angle *θ* varied from 0° to 20°. Under normal incidence (*θ =* 0°), the metasurface exhibits strong attenuation of RCP light while remaining largely transmissive for LCP light resulting in a pronounced polarization contrast. This behavior originates from the intrinsic chirality of the asymmetric gammadion geometry, which enables selective excitation of high‐order multipolar resonances at this wavelength. As the incident angle increases, the evolution of the transmittance differs for the two helicities indicating that the underlying multipolar resonances respond differently to oblique excitation depending on the polarization state. This helicity‐dependent angular dispersion reflects the fact that the dominant resonant modes at 652 nm (primarily the Q_e_‐ and Q_m_‐dominated modes identified in Figure 2) retain strong polarization selectivity even when the illumination direction deviates from normal incidence. The corresponding angle‐resolved CD_abs_ map in Figure 4C further highlights this behavior. The CD_abs_ reaches a peak value exceeding 52% near normal incidence and remains above 40% over a broad angular range up to ∼13°. Such sustained CD_abs_ under oblique illumination demonstrates that the chiroptical response is not limited to a narrow angular condition but is instead intrinsically robust. This robustness can be attributed to the combined effects of intrinsic chirality, arising from the broken in‐plane mirror symmetry of the metasurface, and extrinsic chirality introduced by the tilted incident wave vector. Figure 4D,E present the corresponding angle‐resolved transmittance maps for the secondary resonance near 735 nm. In contrast to the behavior observed at 652 nm, the transmittance responses under RCP and LCP illumination in this spectral region become much more symmetric across the entire angular range up to 20°. Consequently, the CD_abs_ map in Figure 4F exhibits a comparatively weaker CD_abs_ response with a maximum value of ∼42%. This reduced CD_abs_ magnitude under normal incidence can be attributed to the involvement of fewer and less polarization‐selective multipolar modes at this wavelength. As shown in Figure 2, both RCP and LCP illumination at 735 nm excite largely overlapping sets of multipolar resonances resulting in a smaller imbalance in modal excitation. Notably, the reduced complexity of multipolar coupling at 735 nm also leads to a CD_abs_ response that is less sensitive to variations in the incident angle. Because fewer multipolar channels contribute to the chiroptical response, the interference conditions governing CD_abs_ are less perturbed under oblique illumination. As a result, the CD_abs_ remains relatively stable across a wide angular range despite its smaller magnitude compared to the primary resonance. This comparison between the two resonances highlights a trade‐off between CD_abs_ strength and angular sensitivity: while strong CD_abs_ at 652 nm arises from highly polarization‐selective excitation of multiple high‐order modes, the more moderate yet angle‐insensitive CD_abs_ at 735 nm originates from a simpler and more symmetric multipolar excitation landscape.

**FIGURE 4:**
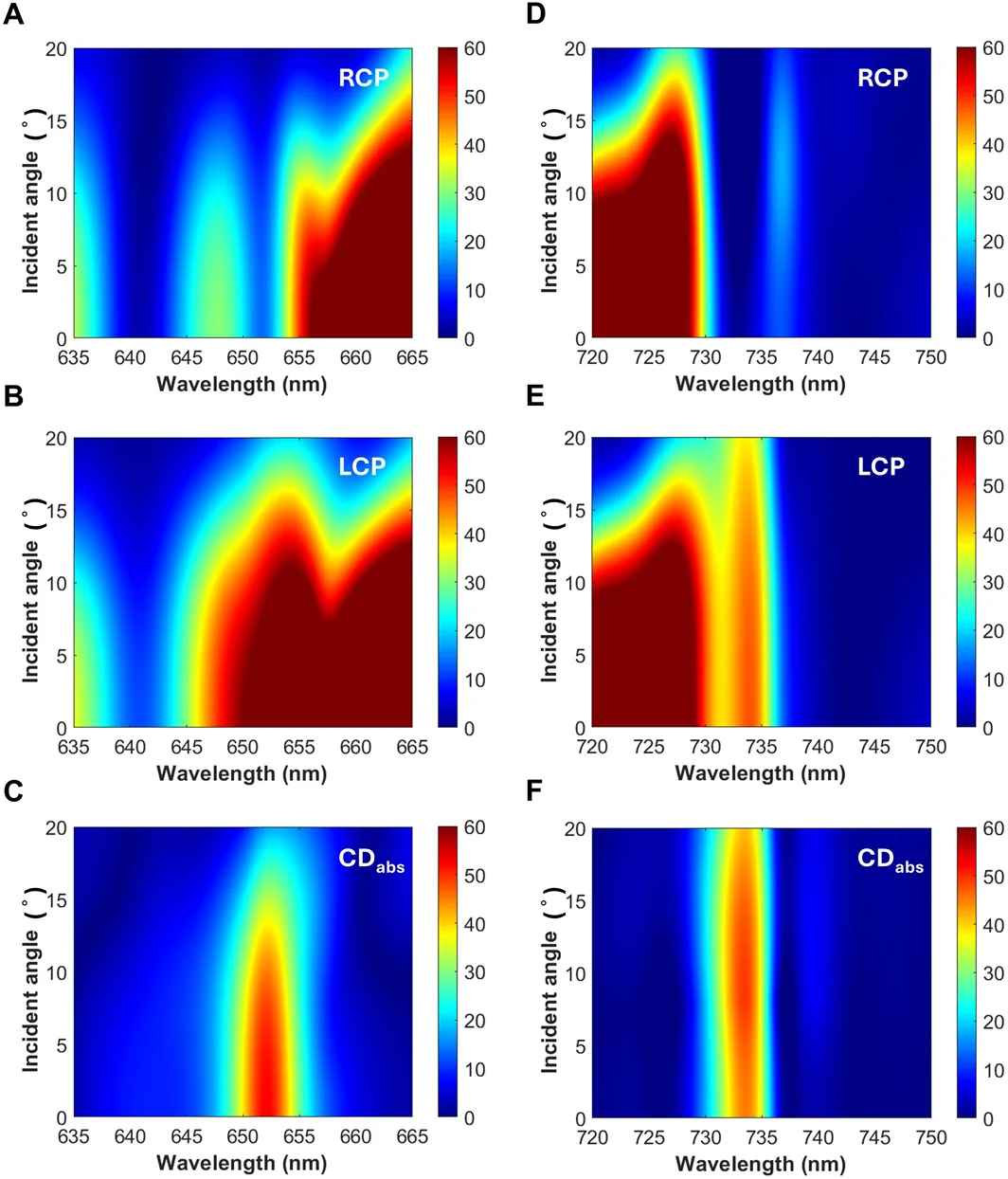
Angle‐resolved transmittance and absolute circular dichroism CD_abs_ of the all‐dielectric asymmetric gammadion metasurface. (A, B) Simulated transmittance maps under RCP and LCP illumination, respectively, as functions of wavelength and incident angle *θ* in the spectral region around the primary CD_abs_ resonance near 652 nm. (C) Corresponding angle‐resolved CD_abs_ map calculated from the difference between the RCP and LCP transmittance. (D, E) Simulated transmittance maps under RCP and LCP illumination, respectively, in the longer‐wavelength resonance region near 735 nm for incident angles up to 20°. (F) Corresponding CD_abs_ map for the 735 nm resonance.

## 3 Conclusion

In summary, we have demonstrated an all‐dielectric asymmetric gammadion metasurface that exhibits pronounced and angle‐robust CD_abs_ in the visible spectral range. By deliberately breaking the in‐plane mirror symmetry of a high‐index a‐Si metasurface, strong polarization‐dependent light–matter interactions are enabled, allowing the conversion of structural chirality into measurable far‐field optical chirality. Numerical simulations and experimental results consistently reveal two prominent CD_abs_ resonances near 652 and 735 nm, whose amplitudes and spectral positions can be tuned through structural asymmetry. Through a comprehensive electromagnetic multipole decomposition, we have elucidated the physical origins of these chiroptical responses. At the primary resonance near 652 nm, the CD_abs_ is governed by the polarization‐selective excitation of high‐order multipolar modes, particularly electric and magnetic quadrupoles, which are strongly excited under RCP illumination but suppressed under LCP illumination. This pronounced asymmetry in the multipolar scattering landscape gives rise to a large differential far‐field response and is directly responsible for the observed high CD_abs_. In contrast, the secondary resonance near 735 nm involves fewer and more symmetric multipolar contributions under opposite circular polarizations leading to a reduced but still observable CD_abs_ response. Near‐field analysis further reveals that the selective excitation of high‐order multipolar modes generates strongly localized chiral near fields. The electric and magnetic quadrupole dominated resonances produce spatially confined regions of enhanced optical chirality, resulting in a large differential total optical chirality enhancement between RCP and LCP illumination. The strong correspondence between multipolar field distributions, localized optical chirality, and far‐field CD_abs_ establishes a clear physical link between modal excitation and chiroptical response. A route suggested by our additional loss reduction test for further CD enhancement is to employ a lower‐loss dielectric platform or reduce the material extinction coefficient, which improves the resonance quality and strengthens the helicity‐selective interference. In addition, more complex geometries with additional degrees of freedom, such as coupled or multilayer resonators, could further suppress the undesired‐helicity transmission channel and improve the overall CD_abs_ response. In this sense, our present design was intended primarily to elucidate the distinct multipolar origins of the two visible CD_abs_ resonances in a simple asymmetric a‐Si metasurface rather than to maximize the absolute peak CD_abs_. We note that although the metasurface is not deeply subwavelength over the entire spectral range, additional diffraction efficiency calculations show that the first higher diffraction orders are weak (< 2%) and only appear below ∼500 nm (see Supporting Information S1: Figure S7). Therefore, the CD_abs_ resonances discussed here are dominated by the zeroth‐order transmitted fields. Angle‐resolved studies demonstrate that the metasurface maintains robust chiroptical performance under oblique incidence. The strong CD_abs_ at 652 nm persists over a wide angular range due to the resilience of polarization‐selective quadrupolar modes supported by the combined effects of intrinsic and extrinsic chirality. Meanwhile, the weaker CD_abs_ at 735 nm, arising from a simpler multipolar excitation landscape, exhibits reduced sensitivity to the incident angle highlighting a trade‐off between CD_abs_ magnitude and angular stability. These findings underscore the critical role of multipolar mode engineering in tailoring both the strength and robustness of chiroptical responses. The insights presented here offer clear design guidelines for developing low‐loss polarization‐selective nanophotonic devices and pave the way toward advanced applications in chiral sensing, polarization optics, and integrated photonic systems.

## 4 Methods

### 4.1 Simulation

The computational analyses were performed using the COMSOL Multiphysics commercial software suite, which enables precise simulation of field distributions, multipole decomposition, and reflection behavior. The design of the asymmetric gammadion metasurface was numerically approached using the wave optics module. For the calculation of the single meta‐atom design, a unit cell boundary condition was applied along the *x*‐direction and *y*‐direction to simulate transmission and phase shift in an array structure. Floquet periodic boundary conditions were set along the *x* and *y* axes for normal and oblique incidence. Similarly, the distributions of various electromagnetic fields on each structural cross‐section can also be revealed through the simulation results. To determine the scattering cross‐section, a perfectly matched layer (PML) was utilized in the simulations. The refractive indices of a‐Si and SiO_2_ were determined through ellipsometry measurements.

## Author Contributions

**Chien‐Feng Chien:** writing – original draft, data curation, investigation. **Hsiu‐Ping Su:** conceptualization, supervision, validation. **Yu‐Tsung Lin:** validation, supervision. **Shu‐Fei Ho:** methodology, data curation. **Pin Chieh Wu:** conceptualization, supervision, writing – original draft, funding acquisition, investigation.

## Conflicts of Interest

The authors declare no conflicts of interest.

## Supporting information


Supporting Information S1

## Data Availability

The data that support the findings of this study are available from the corresponding author upon reasonable request.
