# Supplementary material for: High‐Order Multipolar Excitations Governing Circular Dichroism in an All‐Dielectric Chiral Metasurface
Source: Nanophotonics. 2026 May 28;15(11):e70153. doi: 10.1002/nap2.70153 (PMC13642064; doi:10.1002/nap2.70153)
Supplement: Supplementary file 1 [file NAP2-15-e70153-s001.pdf]

Supplementary Material

High-order multipolar excitations governing circular dichroism in an all-dielectric chiral metasurface

Chien-Feng Chien,[1] Hsiu-Ping Su,[1] Yu-Tsung Lin,[1] Shu-Fei Ho[1], and Pin Chieh Wu[1,2,3*]

[1]Department of Photonics, National Cheng Kung University, Tainan 70101, Taiwan
[2]Center for Quantum Frontiers of Research & Technology (QFort), National Cheng Kung University, Tainan 70101, Taiwan
[3]Meta-nanoPhotonics Center, National Cheng Kung University, Tainan 70101, Taiwan

*E-mail: pcwu@gs.ncku.edu.tw

Electromagnetic multipole expansion

We employ the electromagnetic multipole expansion[1, 2] to assess each electromagnetic mode and its impact on the far-field radiation. Table S1 below displays the equations for integrating the displacement current (*J*) of the dielectric medium within the metasurface unit cell. The symbols *p*, *m*, and T correspond to electric dipole, magnetic dipole, and toroidal dipole, respectively. $Q^{(e)}$, $Q^{(m)}$, $Q^{(T)}$, $O^{(e)}$, $O^{(m)}$, and $O^{(T)}$ indicate the electric quadrupole, magnetic quadrupole, toroidal quadrupole, electric octupole, magnetic octupole and toroidal octupole. Here, *c* is the speed of light in vacuum, *ω* is the angular frequency.

Table S1

Type	Multipoles	
Electric dipole	$p_\alpha = \frac{1}{i\omega}\int J_\alpha(r)d^3r$	Eq. (S1)
Magnetic dipole	$m_\alpha = \frac{1}{2c}\int [r \times J(r)]_\alpha d^3r$	Eq. (S2)
Toroidal dipole	$T_\alpha^{(e)} = \frac{1}{10c}\int \left[\left(r \cdot J(r)\right)r_\alpha - 2r^2 J_\alpha(r)\right]d^3r$	Eq. (S3)
Electric quadrupole	$Q_{\alpha,\beta}^{(e)} = \frac{1}{2i\omega}\int \left[r_\alpha J_\beta(r) + r_\beta J_\alpha(r) - \frac{2}{3}\delta_{\alpha,\beta}\left(r \cdot J(r)\right)\right] d^3r$	Eq. (S4)
Magnetic quadrupole	$Q_{\alpha,\beta}^{(m)} = \frac{1}{3c}\int \left[[r \times J(r)]_\alpha r_\beta\right]d^3r + \{\alpha \leftrightarrow \beta\}$	Eq. (S5)
Toroidal quadrupole	$Q_{\alpha,\beta}^{(T)} = \frac{1}{28c}\int \left[4r_\alpha r_\beta\left(r \cdot J(r)\right) - 5r^2\left(r_\alpha J_\beta(r) + r_\beta J_\alpha(r)\right) + 2r^2\left(r \cdot J(r)\right)\delta_{\alpha,\beta}\right] d^3r$	Eq. (S6)
Electric octupole	$O_{\alpha,\beta,\gamma}^{(e)} = \frac{1}{6}\int \left[\rho r_\alpha\left(\frac{r_\beta r_\gamma}{3} - \frac{1}{5}r^2\delta_{\beta,\gamma}\right)\right] d^3r + \{\alpha \leftrightarrow \beta,\gamma\} + \{\alpha \leftrightarrow \gamma,\beta\}$	Eq. (S7)
Magnetic octupole	$O_{\alpha,\beta,\gamma}^{(m)} = \frac{15}{2c}\int \left(r_\alpha r_\beta - \frac{1}{5}r^2\delta_{\alpha,\beta}\right) \cdot [r \times J(r)]_\gamma d^3r + \{\alpha \leftrightarrow \beta,\gamma\} + \{\alpha \leftrightarrow \gamma,\beta\}$	Eq. (S8)

Toroidal octupole	$O^{(T)}_{\alpha,\beta,\gamma} = \frac{1}{300}\int 35(r\cdot J(r))r_\alpha r_\beta r_\gamma - 20r^2(J_l r_k r_j + J_k r_l r_k + J_j r_l r_k) + (\delta_{l,j}\delta_{k,p} + \delta_{\gamma,\beta}\delta_{j,p} + \delta_{l,p}\delta_{j,k})[(r\cdot J(r))r^2 r_p + 4J_p r^4]d^3r + \{\alpha \leftrightarrow \beta,\gamma\} + \{\alpha \leftrightarrow \gamma,\beta\}$	Eq. (S9)

where α, β, $\gamma = x, y, z$

Table S2

Type	Scattered intensity	
Electric dipole	$I_{p_a} = \frac{2\omega^4}{3c^3}\ \lvert p_a\rvert^2$	Eq. (S10)
Magnetic dipole	$I_{m_a} = \frac{2\omega^4}{3c^3}\ \lvert m_a\rvert^2$	Eq. (S11)
Toroidal dipole	$I_{T_a} = \frac{2\omega^6}{3c^5}\ \lvert T_a\rvert^2$	Eq. (S12)
Electric quadrupole	$I_{Q^{(e)}_{\alpha,\beta}} = \frac{\omega^6}{180c^5}\sum\left\lvert Q^{(e)}_{\alpha,\beta}\right\rvert^2$	Eq. (S13)
Magnetic quadrupole	$I_{Q^{(m)}_{\alpha,\beta}} = \frac{\omega^6}{180c^5}\sum\left\lvert Q^{(m)}_{\alpha,\beta}\right\rvert^2$	Eq. (S14)
Toroidal quadrupole	$I_{Q^{(T)}_{\alpha,\beta}} = \frac{\omega^8}{180c^7}\sum\left\lvert Q^{(T)}_{\alpha,\beta}\right\rvert^2$	Eq. (S15)
Electric octupole	$I_{O^{(e)}_{\alpha,\beta,\gamma}} = \frac{2\omega^6}{945c^5}\sum\left\lvert O^{(e)}_{\alpha,\beta}\right\rvert^2$	Eq. (S16)
Magnetic octupole	$I_{O^{(m)}_{\alpha,\beta,\gamma}} = \frac{2\omega^6}{945c^5}\sum\left\lvert O^{(m)}_{\alpha,\beta,\gamma}\right\rvert^2$	Eq. (S17)
Toroidal octupole	$I_{O^{(T)}_{\alpha,\beta,\gamma}} = \frac{2\omega^8}{945c^7}\sum\left\lvert O^{(T)}_{\alpha,\beta,\gamma}\right\rvert^2$	Eq. (S18)

where α, β, $\gamma = x, y, z$

The scattered intensity for each electromagnetic multipole is provided in Table S2. Accordingly, the radiation intensity of the scattered field can be represented as follows [3]:

$$I_{scattered} \cong \frac{1}{8\pi\varepsilon_0}\left(\frac{2\omega^4}{3c^3}\ \lvert p_a\rvert^2 + \frac{2\omega^4}{3c^3}\ \lvert m_a\rvert^2 + \frac{2\omega^6}{3c^5}\ \lvert T_a\rvert^2 + \frac{\omega^6}{180c^5}\Sigma\left\lvert Q^{(e)}_{\alpha,\beta}\right\rvert^2 + \frac{\omega^6}{180c^5}\Sigma\left\lvert Q^{(m)}_{\alpha,\beta}\right\rvert^2 + \frac{\omega^8}{180c^7}\Sigma\left\lvert Q^{(T)}_{\alpha,\beta}\right\rvert^2 + + \frac{2\omega^6}{945c^5}\Sigma\left\lvert O^{(e)}_{\alpha,\beta,\gamma}\right\rvert^2 + \frac{2\omega^6}{945c^5}\Sigma\left\lvert O^{(e)}_{\alpha,\beta,\gamma}\right\rvert^2 + \frac{2\omega^8}{945c^7}\Sigma\left\lvert O^{(T)}_{\alpha,\beta,\gamma}\right\rvert^2\right) \qquad \text{Eq. (S19)}$$

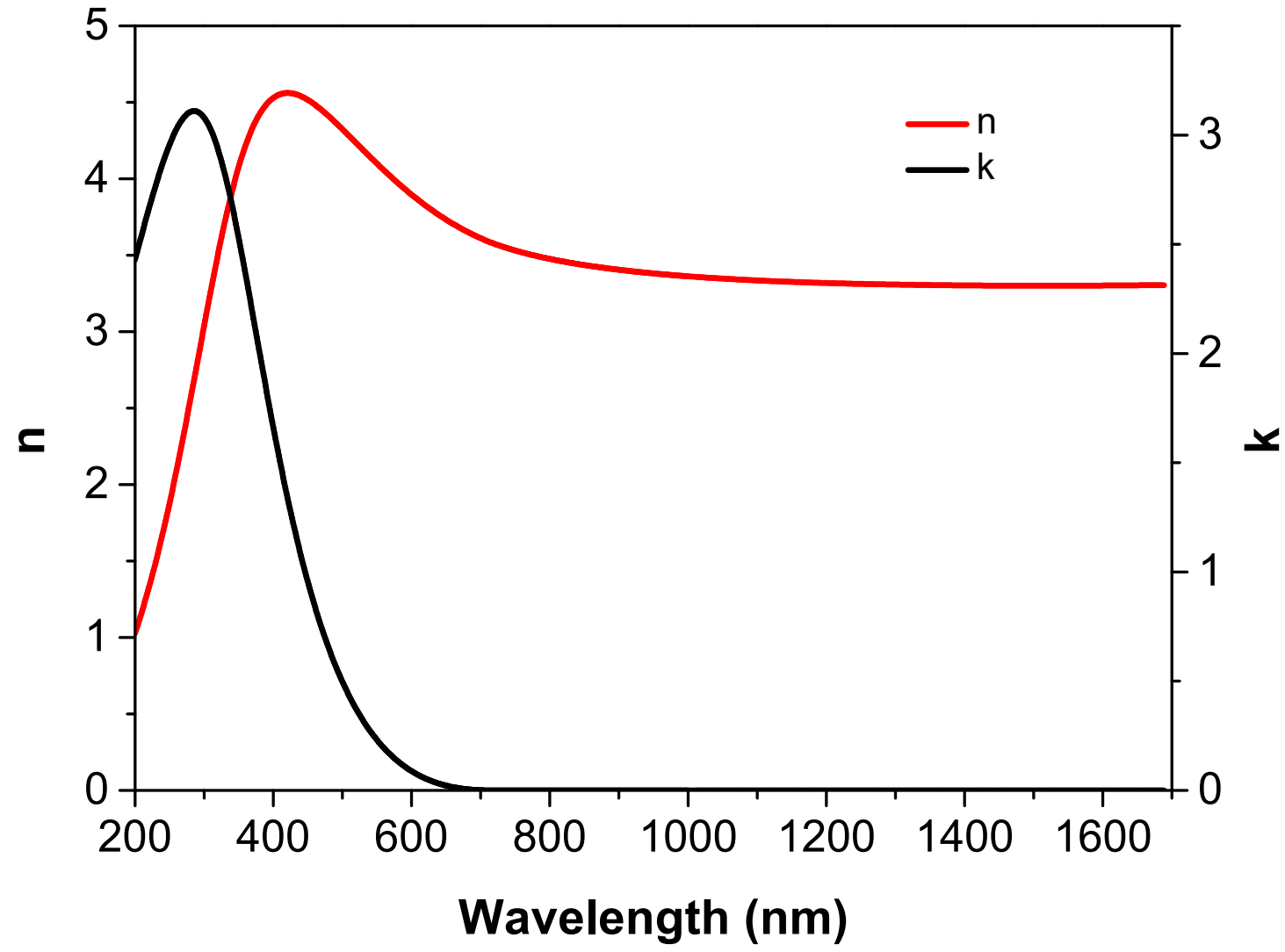

Figure S1. Refractive index (n) and extinction coefficient (k) of a-Si measured through ellipsometry.

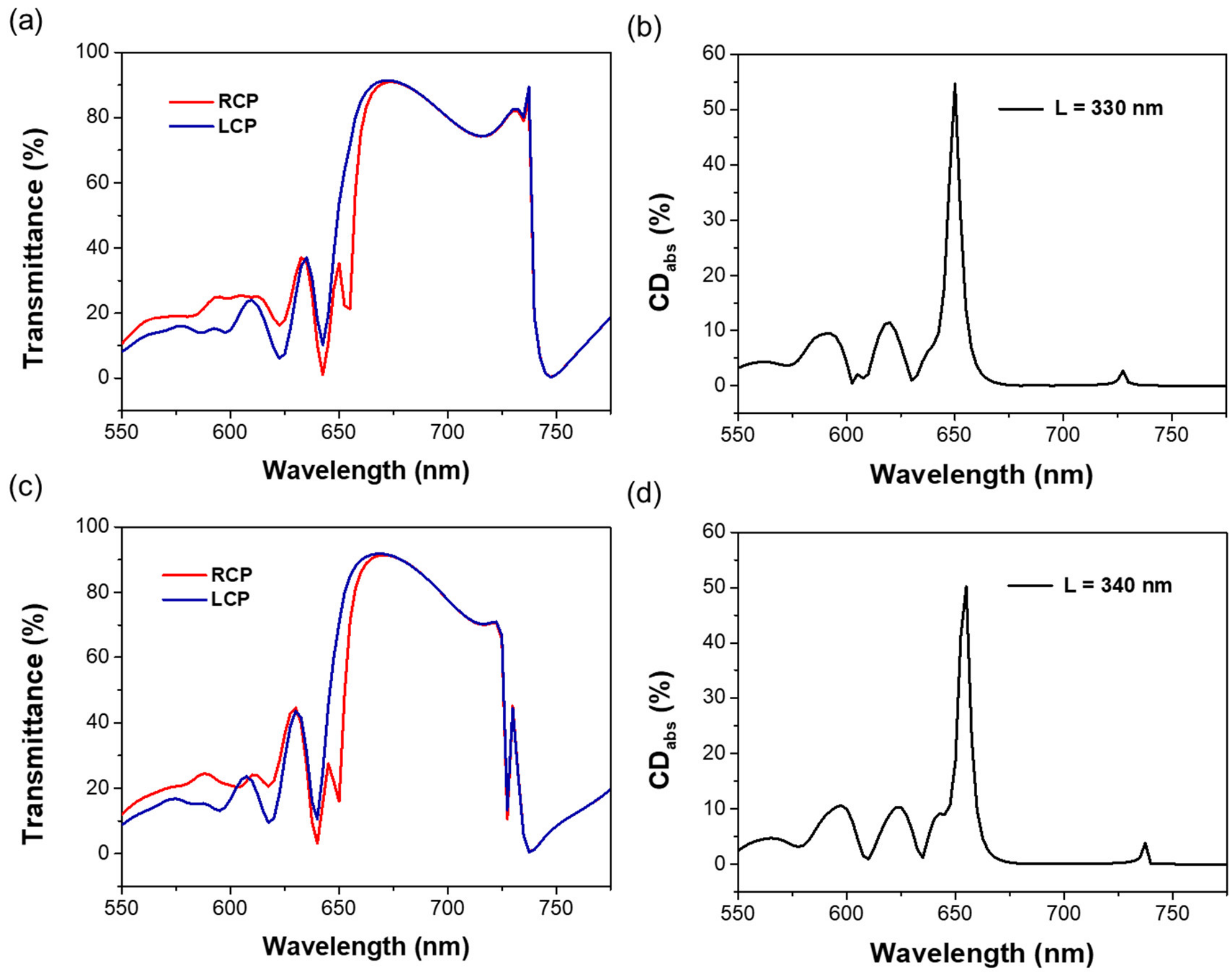

Figure S2. (A, C) Simulated co-polarized transmittance under normal incidence for RCP and LCP light in the wavelength range of 550-775 nm. (B, D) Corresponding absolute circular dichroism (CD_{abs}) distributions. Here, $L_1 = L_2 = L$, indicating that the metasurface is designed with a C_4 symmetry.

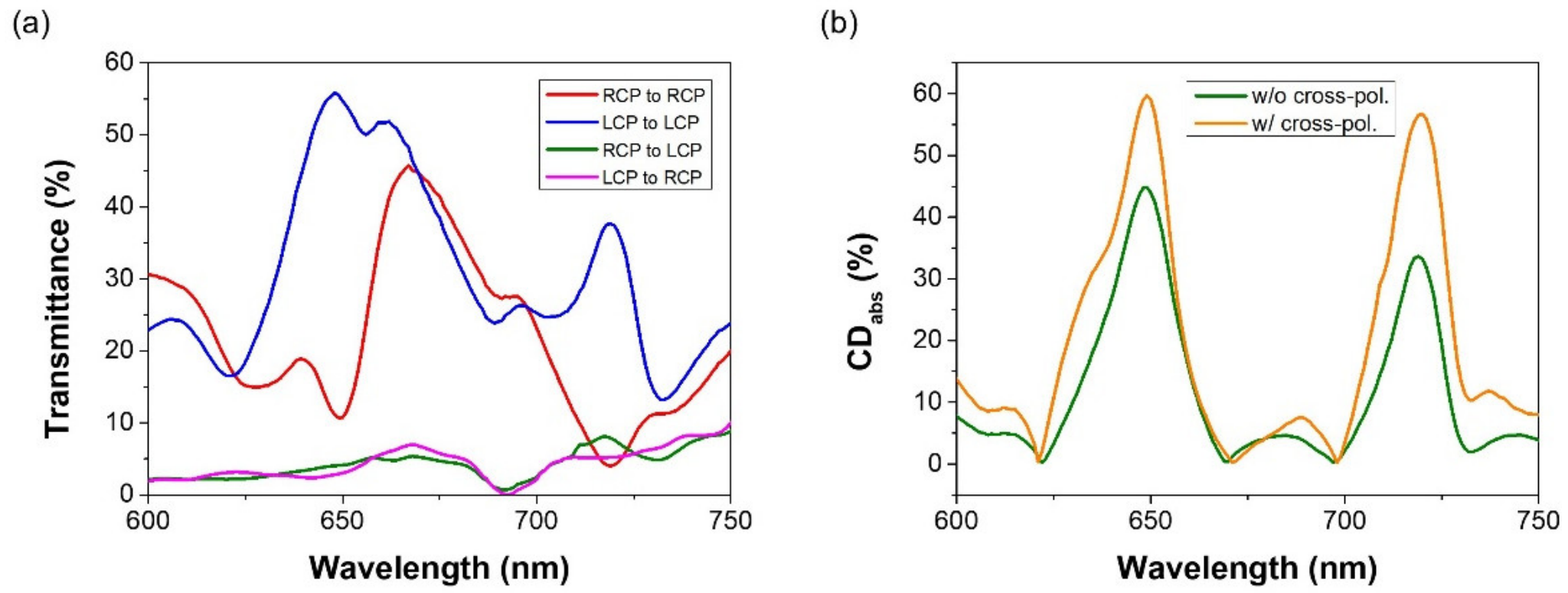

Figure S3. (a) Measured co-polarized and cross-polarized transmittance spectra under circularly polarized incidence, including RCP-to-RCP, LCP-to-LCP, RCP-to-LCP, and LCP-to-RCP channels. The cross-polarized components remain much weaker than the co-polarized components over the wavelength range of interest. (b) Comparison between the experimentally extracted CD_{abs} spectra calculated without and with inclusion of the cross-polarized transmittance components. When the cross-polarized transmission components are included, the $CD_{abs,cross}$ is defined as: $\mathrm{CD}_{\mathrm{abs,cross}} = \left|\frac{(T_{lr}+T_{rr})-(T_{rl}+T_{ll})}{(T_{lr}+T_{rr})+(T_{rl}+T_{ll})}\right|$.

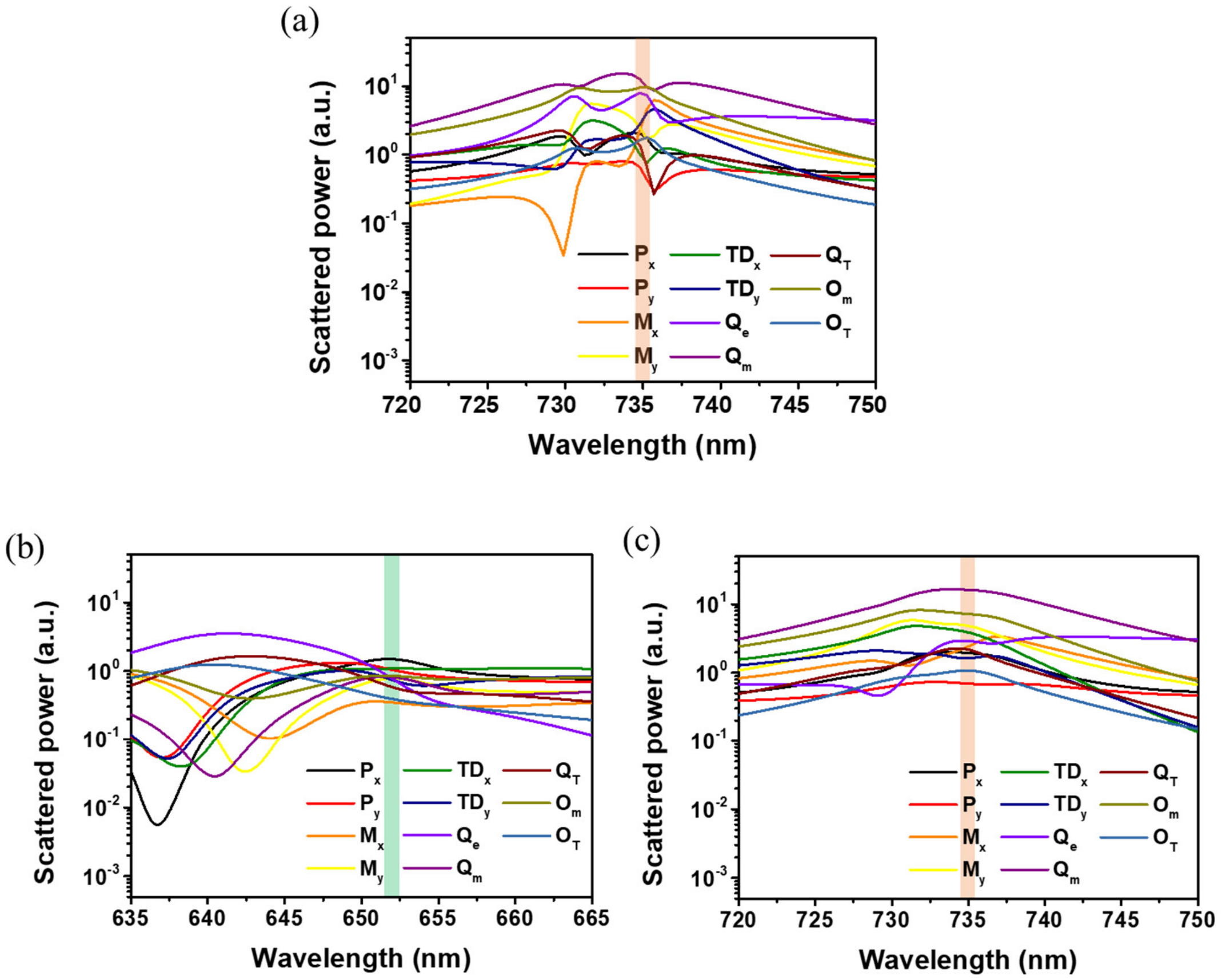

Figure S4. Electromagnetic multipole decomposition of the scattered power from the all-dielectric a-Si asymmetric gammadion metasurface under RCP and LCP illumination. (a) Scattered power distribution under RCP illumination in the wavelength range of 720–750 nm. (b) and (c) display the corresponding distributions under LCP illumination for the spectral ranges of 635-665 nm and 720-750 nm, respectively.

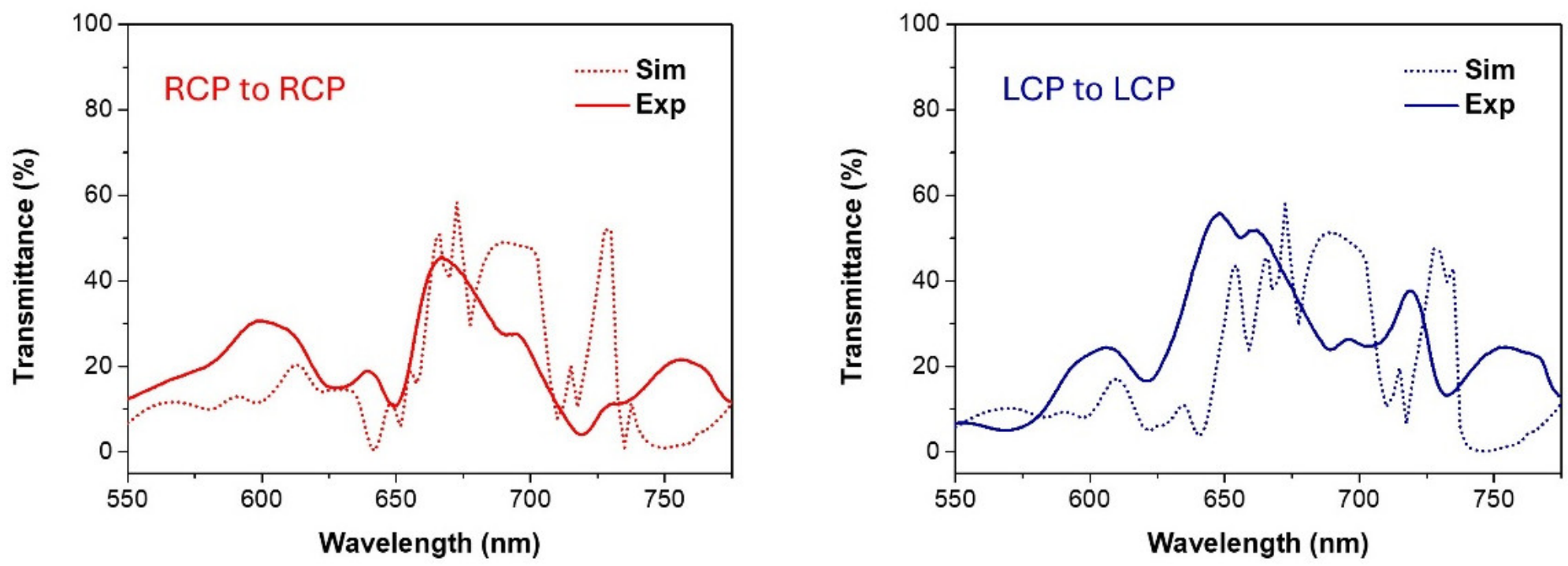

Figure S5. Simulated and experimentally measured transmission spectra of the metasurface under RCP and LCP illumination, respectively. The experimental data are taken from Fig. 1 of the main text, while the simulated spectra are calculated for an oblique incidence angle of 15°.

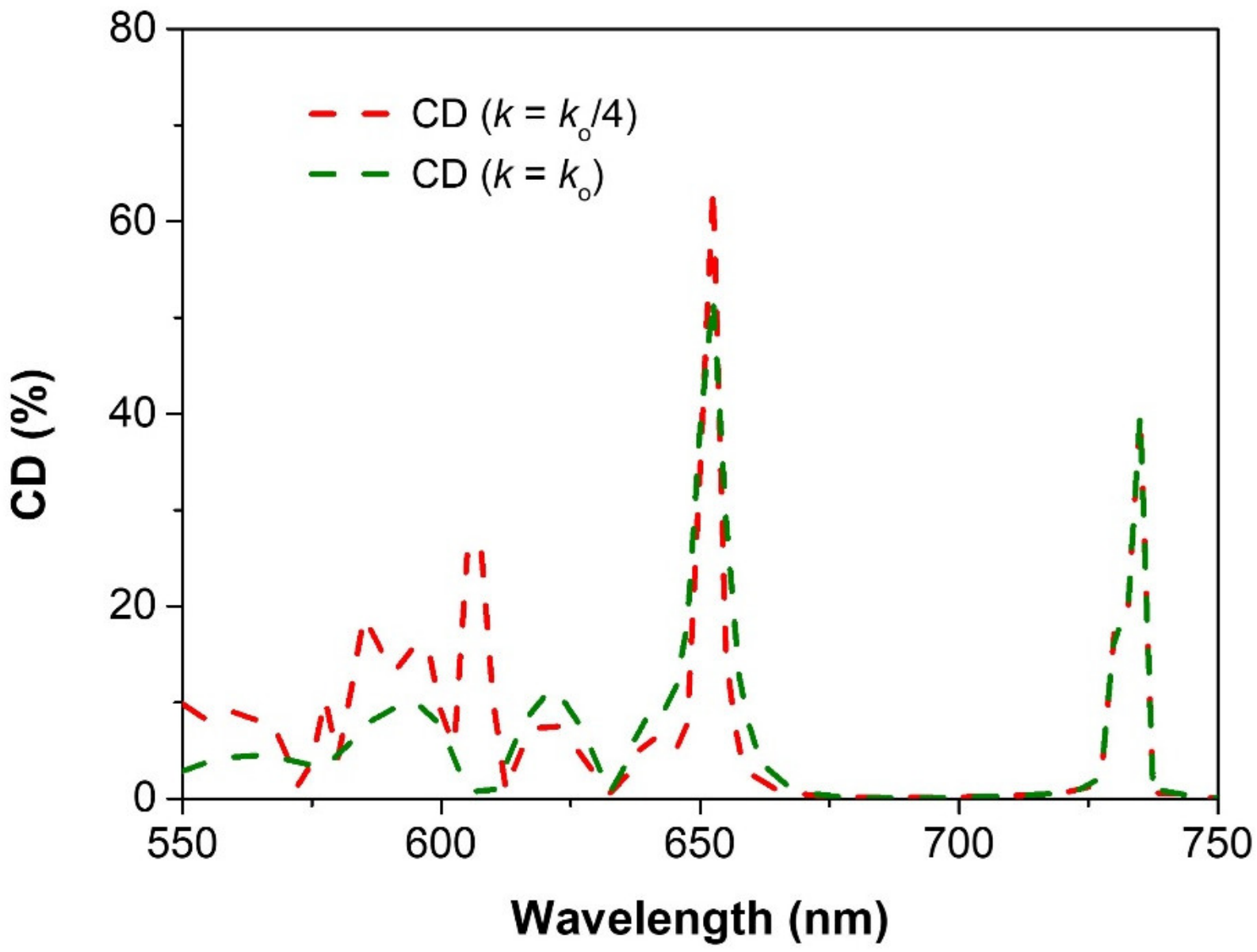

Figure S6. Simulated CD spectra of the metasurface with different extinction coefficient. k_0 represents the original extinction coefficient experimentally obtained from ellipsometry measurement.

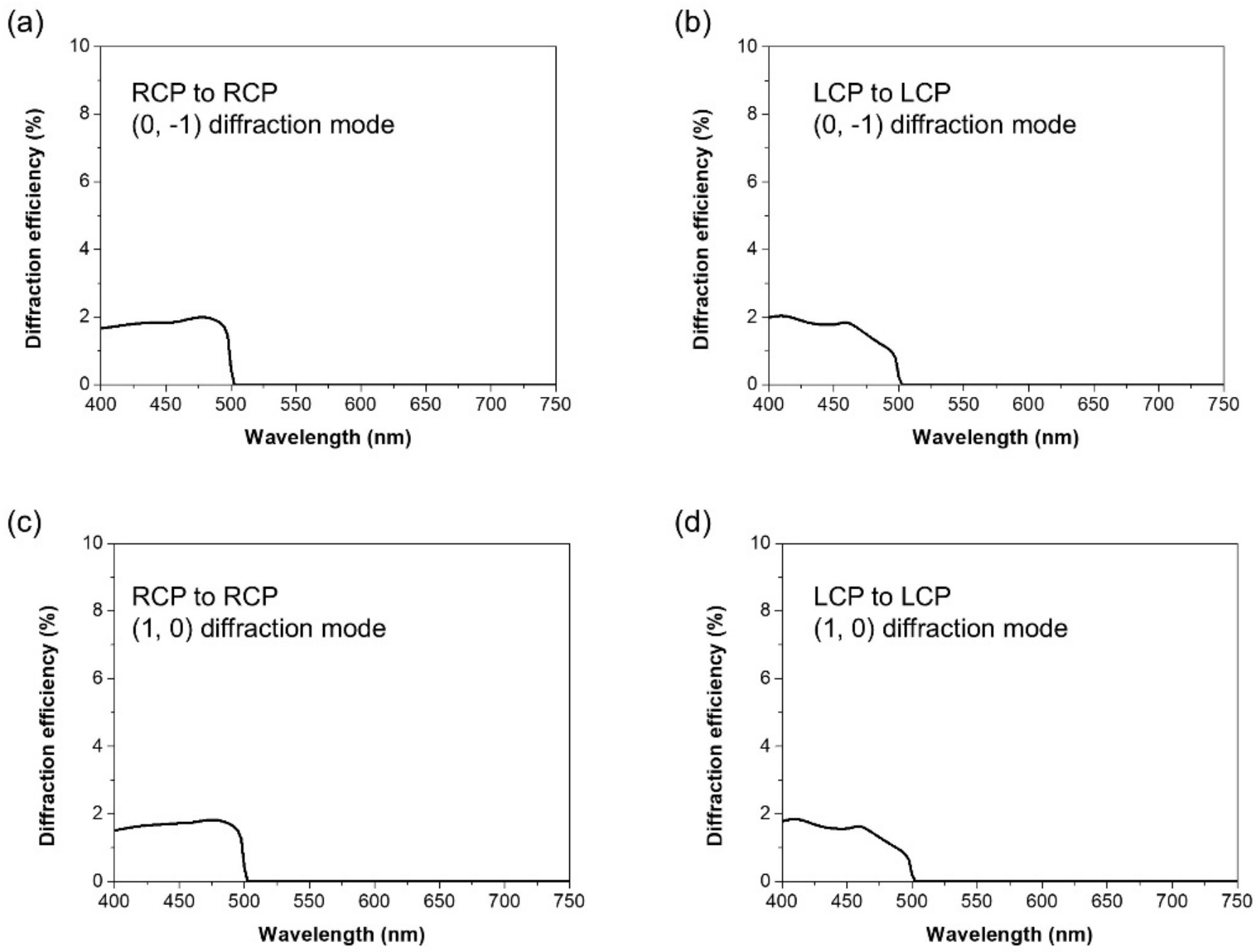

Figure S7. Simulated diffraction efficiencies of the metasurface for the co-polarized transmitted channels in the first higher diffraction orders: (a) RCP-to-RCP and (b) LCP-to-LCP for the (0, -1) order, and (c) RCP-to-RCP and (d) LCP-to-LCP for the (1, 0) order.

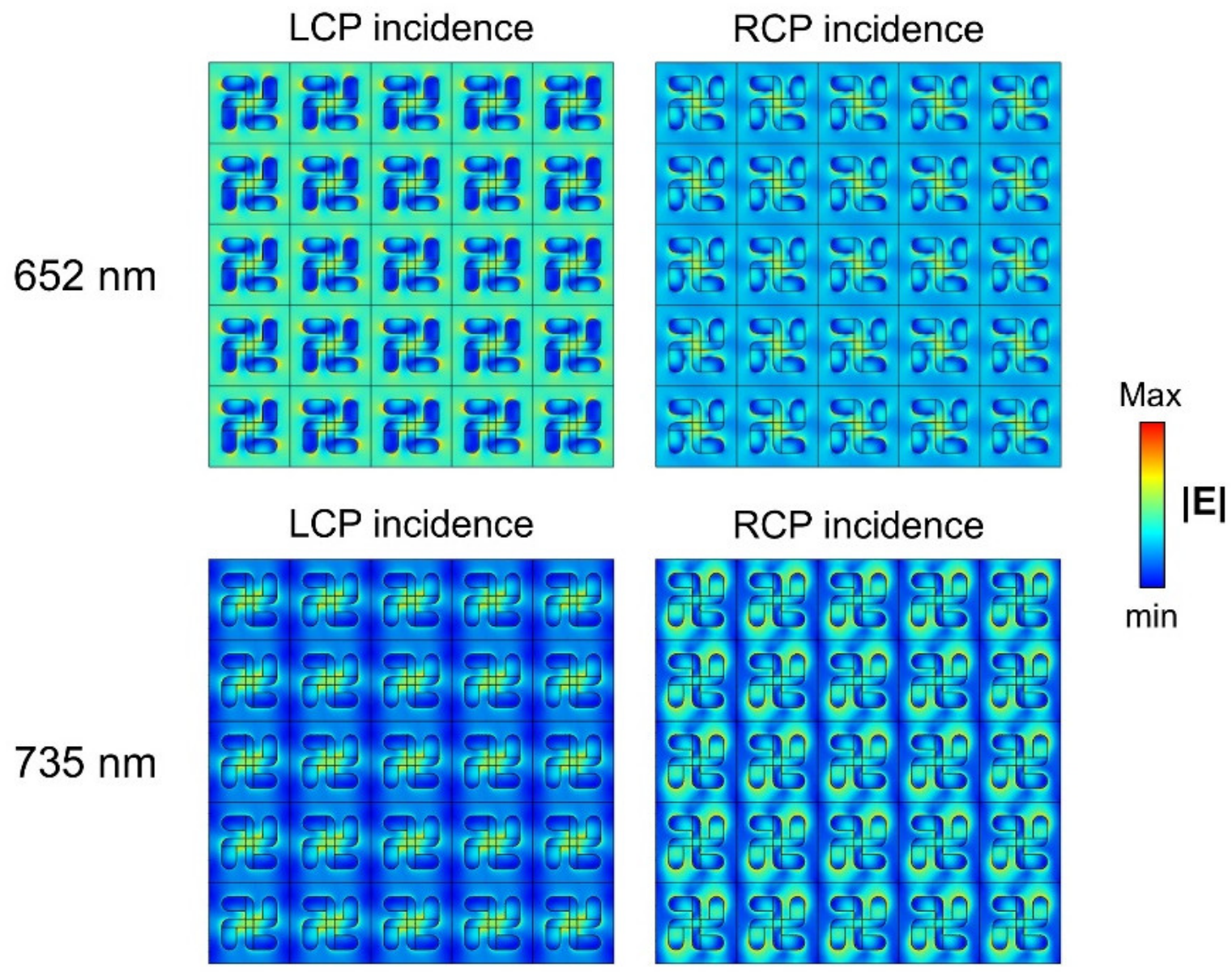

Figure S8. Simulated electric field magnitude distributions of a metasurface array under LCP and RCP illumination at the two CD resonance wavelengths.

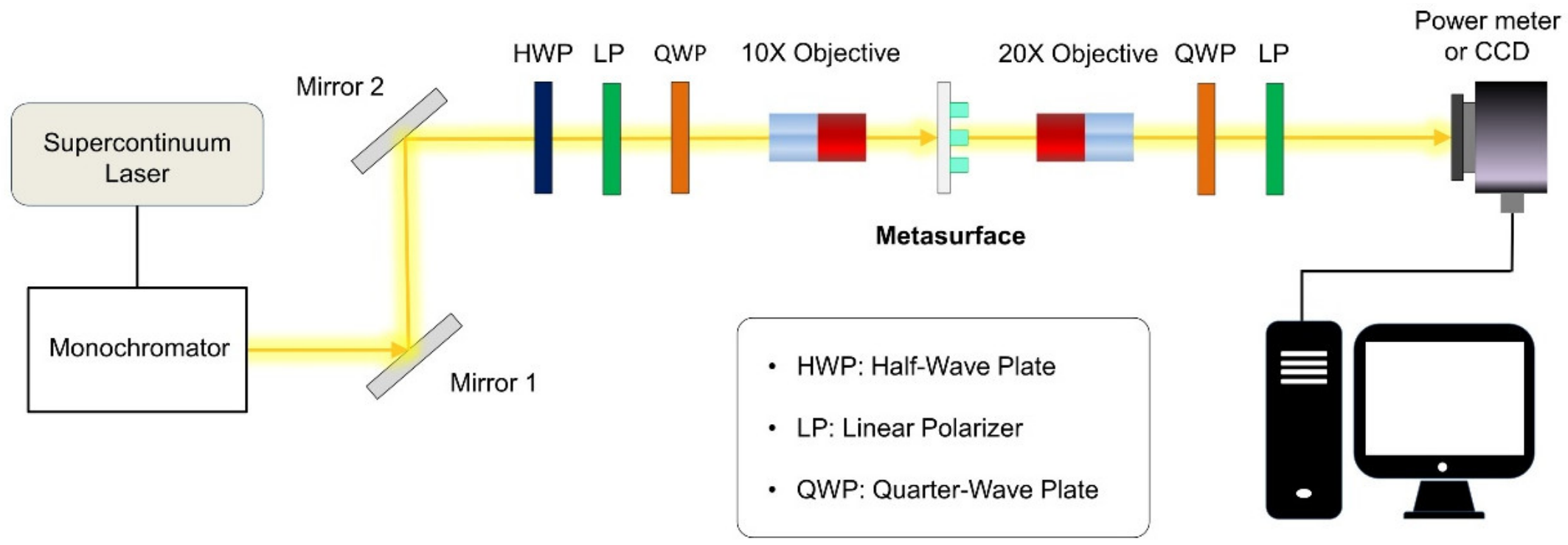

Figure S9. Optical setup for spectrum measurements. A supercontinuum laser (NKT Photonics FIU-15) combined with a monochromator is utilized to select the wavelength in the visible. A half-wave plate (HWP), a linear polarizer (LP), and a quarter-wave plate (QWP) are used to determine the polarization state. A 10× objective (NA = 0.26) is used to focus the incident laser onto the metasurface. The detector is either a power meter (for intensity measurement) or a CCD (for sample imaging).

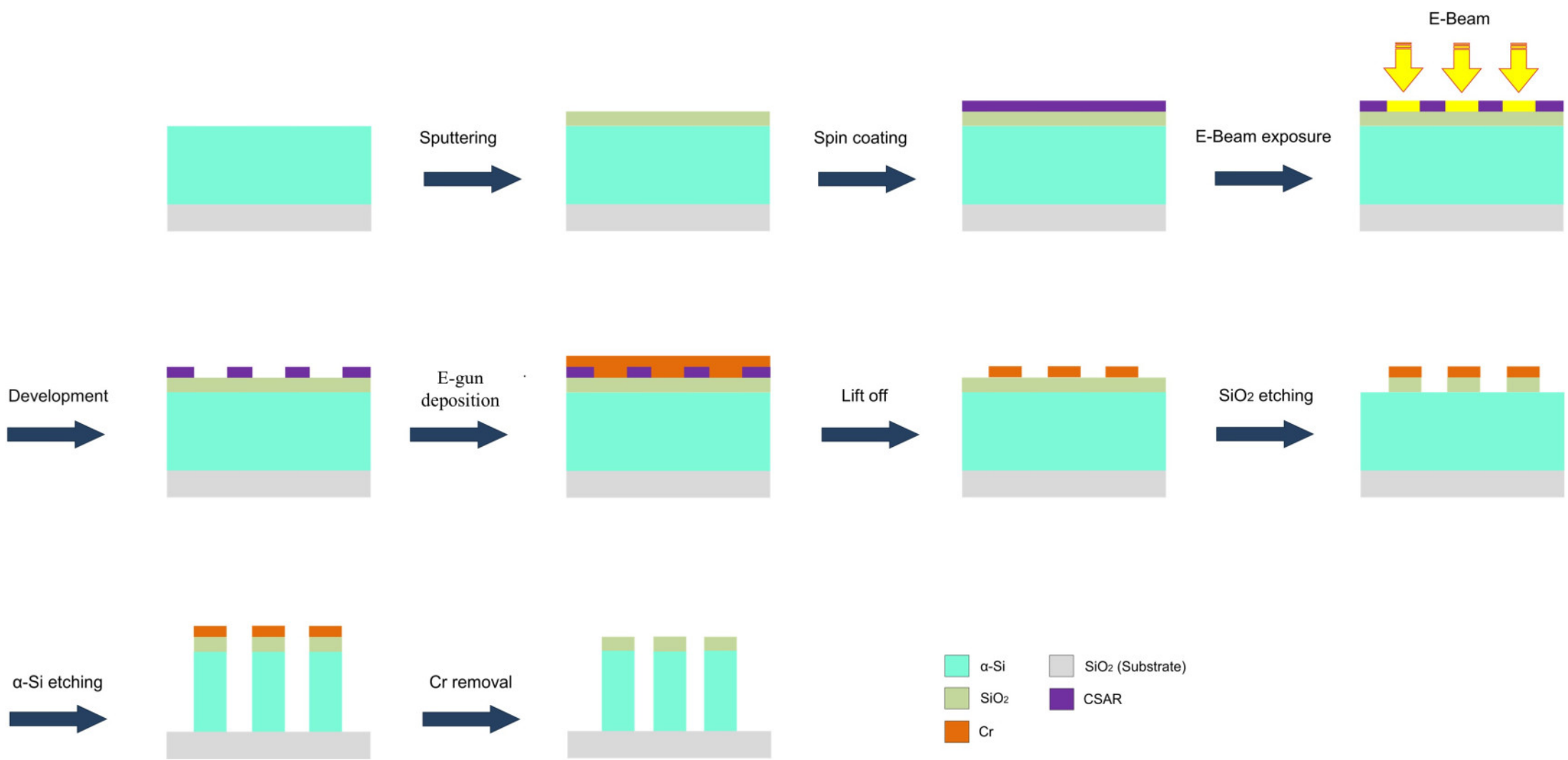

Figure S10. Fabrication flow for the a-Si metasurface. A SiO_2 thin film was first deposited on the substrate by sputtering. A CSAR e-beam resist was then spin-coated on the pre-prepared substrate, followed by e-beam lithography to define the metasurface pattern. After development, a Cr hard mask was deposited by e-gun evaporation, and the lift-off process was carried out to leave the patterned Cr mask on the sample surface. The pattern was subsequently transferred into the underlying a-Si layer by dry etching processes, and the remaining Cr mask was finally removed, yielding the fabricated a-Si metasurface structures.

References

[1] E. A. Gurvitz, K. S. Ladutenko, P. A. Dergachev, A. B. Evlyukhin, A. E. Miroshnichenko, and A. S. Shalin, "The high-order toroidal moments and anapole states in all-dielectric photonics," *Laser Photon. Rev.*, vol. **13**, no. 5, pp. 1800266 (2019).

[2] A. Hassanfiroozi, P. S. Huang, S. H. Huang, K. I. Lin, Y. T. Lin, C. F. Chien, Y. Shi, W. J. Lee, and P. C. Wu, "A toroidal-Fano-resonant metasurface with optimal cross-polarization efficiency and switchable nonlinearity in the near-infrared," *Adv. Opt. Mater.*, vol. **9**, no. 21, pp. 2101007 (2021).

[3] S. Shen, Z. Ruan, S. Li, Y. Yuan, and H. Tan, "The influence of periodicity on the optical response of cube silicon metasurfaces," *Results Phys.*, vol. **23**, no. 104057 (2021).
